# Sorcin in Cancer Development and Chemotherapeutic Drug Resistance

**DOI:** 10.3390/cancers16162810

**Published:** 2024-08-09

**Authors:** Cécile Exertier, Lorenzo Antonelli, Annarita Fiorillo, Roberta Bernardini, Beatrice Colotti, Andrea Ilari, Gianni Colotti

**Affiliations:** 1 Institute of Molecular Biology and Pathology, Italian National Research Council (IBPM-CNR), c/o Department Biochemical Sciences, Sapienza University of Rome, Ed. CU027, P.le A.Moro 5, 00185 Rome, Italy; cecile.exertier@cnr.it (C.E.); andrea.ilari@cnr.it (A.I.); 2 Department Biochemical Sciences, Sapienza University of Rome, Ed. CU027, P.le A.Moro 5, 00185 Rome, Italy; lorenzo.antonelli@uniroma1.it (L.A.); annarita.fiorillo@uniroma1.it (A.F.); 3 Department of Clinical Sciences and Translational Medicine, University of Rome “Tor Vergata”, Via Montpellier 1, 00133 Rome, Italy; roberta.bernardini@uniroma2.it; 4 Child Neuropsychiatry Unit, Child Neuropsychiatry School, University Hospital of Tor Vergata, Via Montpellier 1, 00133 Rome, Italy; beatrice.colotti@students.uniroma2.eu

**Keywords:** sorcin, calcium homeostasis, tumorigenesis, drug resistance, targeting sorcin

## Abstract

**Simple Summary:**

Sorcin is a protein that helps cells handle calcium. It is often found in high amounts in cancer cells, especially those that are resistant to treatment. Sorcin plays a key role in cancer growth and spread by helping cancer cells avoid the toxic effects of chemotherapy drugs, acting on multiple cellular mechanisms. This review will explore sorcin’s structure and function, its role in cancer, and how we might be able to target it for new treatments.

**Abstract:**

SOluble Resistance-related Calcium-binding proteIN (sorcin) earned its name due to its co-amplification with ABCB1 in multidrug-resistant cells. Initially thought to be an accidental consequence of this co-amplification, recent research indicates that sorcin plays a more active role as an oncoprotein, significantly impacting multidrug resistance (MDR). Sorcin is a highly expressed calcium-binding protein, often overproduced in human tumors and multidrug-resistant cancers, and is a promising novel MDR marker. In tumors, sorcin levels inversely correlate with both patient response to chemotherapy and overall prognosis. Multidrug-resistant cell lines consistently exhibit higher sorcin expression compared to their parental counterparts. Furthermore, sorcin overexpression via gene transfection enhances drug resistance to various chemotherapeutic drugs across numerous cancer lines. Conversely, silencing sorcin expression reverses drug resistance in many cell lines. Sorcin participates in several mechanisms of MDR, including drug efflux, drug sequestering, cell death inhibition, gene amplification, epithelial-to-mesenchymal transition, angiogenesis, and metastasis. The present review focuses on the structure and function of sorcin, on sorcin’s role in cancer and drug resistance, and on the approaches aimed at targeting sorcin.

## 1. Introduction

Cancer is a complex disease arising from the uncontrolled growth of abnormal cells. This aberrant growth is the result of a series of genetic and epigenetic alterations that disrupt normal cellular processes. The transformation of a normal cell into a malignant one is a gradual process involving multiple steps. Initially, genetic mutations occur due to factors such as environmental carcinogens, radiation, or inherited genetic predispositions. These mutations can affect genes involved in cell growth, differentiation, and repair. Over time, these genetic changes accumulate, leading to the activation of oncogenes (genes that promote cell growth) and the inactivation of tumor suppressor genes (genes that inhibit cell growth).

To successfully progress from a benign tumor to a malignant one, cancer cells acquire a set of characteristic capabilities known as cancer hallmarks. These hallmarks, proposed by Hanahan and Weinberg, provide a conceptual framework for understanding the key features of cancer [1,2]. They include the following: (i) Sustaining proliferative signaling: Cancer cells develop mechanisms to stimulate continuous growth and division, often through the activation of growth factor receptors or aberrant signaling pathways; (ii) Evading growth suppressors: Cancer cells inactivate tumor suppressor genes, which normally act as brakes on cell proliferation; (iii) Resisting cell death: Cancer cells become resistant to programmed cell death (apoptosis), allowing them to survive and proliferate; (iv) Enabling replicative immortality: Cancer cells acquire the ability to divide indefinitely, overcoming the normal limits of a cell’s lifespan; (v) Inducing angiogenesis: Cancer cells stimulate the formation of new blood vessels to supply nutrients and oxygen for tumor growth; (vi) Activating invasion and metastasis: Cancer cells acquire the ability to invade surrounding tissues and spread to distant sites, forming secondary tumors; (vii) Deregulating cellular metabolism: Cancer cells reprogram their energy metabolism to support rapid growth and proliferation; (viii) Avoiding immune destruction: Cancer cells develop strategies to evade the immune system and prevent their elimination. These hallmarks are not mutually exclusive and often interact with each other to promote tumor growth and progression.

Traditional chemotherapy works by targeting these rapidly dividing cells. However, over time, tumors can develop various mechanisms to evade drugs. This is known as drug resistance (see Section 5 and Section 6 below).

Sorcin, a highly expressed calcium-binding protein found in the top 3% of expressed proteins in the human body, is a multifaceted protein important in both tumorigenesis and chemotherapeutic drug resistance.

This review will explore the following: (i) sorcin’s structure and activation, (ii) sorcin’s cellular functions, (iii) sorcin overexpression in tumors and cancer cells, and its functions as an oncoprotein, (iv) the molecular mechanisms underlying sorcin’s role in multidrug resistance (MDR), and (v) the approaches aiming at targeting sorcin.

## 2. Sorcin Structure and Activation

Sorcin (SOluble Resistance-related Calcium-binding proteIN) was first identified co-amplified with a drug transporter protein (ABCB1) in cells resistant to chemotherapeutic drugs [3].

The sorcin gene (SRI) is located on chromosome 7, specifically in region 7q21, and extends approximately 21.9 kilobases. Humans have at least four different sorcin isoforms. Isoform A, the most studied form, is a 22 kDa protein with 198 amino acids, encoded by a transcript with eight exons and seven introns (15 kb long). Other isoforms (B, C, and D) are shorter (19 kDa) due to missing parts of the N-terminal or C-terminal domains. Interestingly, some studies refer to 19 kDa forms of sorcin, despite isoform A being the dominant form. Notably, a pseudogene similar to sorcin (*SRIL*) also exists and it is found on chromosome 4 [4].

Sorcin is a relatively recent protein in evolutionary terms, found in vertebrates and, more broadly, in multicellular organisms. The sorcin protein sequence is remarkably similar across different species. For instance, human and mouse sorcin proteins differ by only eight amino acids, most of which are minor variations.

Structurally, sorcin belongs to the penta-EF-hand (PEF) protein family, which also includes calpains, grancalcin, PDCD6, and peflin [5].

EF hands are structural motifs composed of helix-loop-helix structures. A specific 12-amino acid sequence within this motif enables the high-affinity binding of calcium ions (Ca^2+^), forming a distinctive pentagonal bipyramidal arrangement.

Calcium binding acts as a signaling mechanism in many cellular processes. In the absence of calcium, sorcin exists as a homodimer. Each subunit consists of two domains: a flexible N-terminal domain rich in glycine residues (positions 1–32) and a C-terminal calcium-binding domain (SCBD, positions 33–198) containing five EF hands [6,7]. Typically, proteins that bind calcium have an even number of EF hands for optimal structure and function. However, in sorcin, the EF hands are connected by short two-stranded β-sheets, forming pairs (EF1-EF2 and EF3-EF4). EF5, although not paired within a single sorcin subunit, interacts with the EF5 hand from the other subunit in the sorcin homodimer, contributing to the dimer interface [8,9].

**Figure 1:**
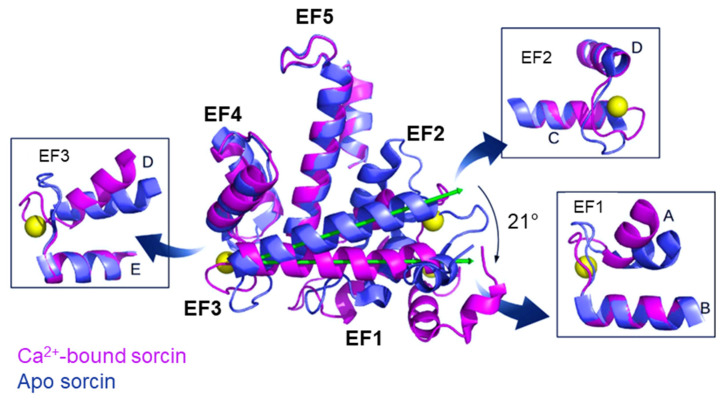
The X-ray crystal structure of human sorcin in the apo form (PDB: 4UPG, blue) and in the calcium-bound form (PDB: 4USL, magenta; calcium ions are represented by yellow spheres). Upon calcium binding, sorcin activation occurs, with a transition from a closed to an open structure, involving a 21° rotation of the long D-helix of 21° and the exposure of hydrophobic surfaces that leads to the interaction with targets. More details of the EF1 hand (part of the A and B helices, and the AB loop), EF2 hand (part of the C and D helices, and the CD loop), and EF3 hand (part of the D and E helices, and the DE loop) are shown in the boxes. Image drawn with the PyMol software (PyMol v.0.99 DeLano Scientific LLC, San Francisco, CA, USA).

Sorcin activation relies on calcium binding to EF1, EF2 and EF3 hands. When Ca^2+^ binds to the EF3 hand, in particular, sorcin undergoes a significant conformational change (Figure 1). This change involves a rotation of the long D-helix connecting the EF1-EF2 subdomain to EF3. Additionally, it opens EF1 and exposes hydrophobic surfaces in the EF1-EF3 region. These structural changes allow sorcin to either aggregate in the absence of protein targets or bind and regulate other proteins in a calcium-dependent manner [6,7,8,9,10,11,12,13,14].

Peptide phage display, a technique used to identify protein–protein interactions, revealed two consensus sequences in proteins that bind to sorcin upon calcium binding. These sequences conform to a Φ/Gly/Met-Φ/Gly/Met-x-P motif (where Φ is an aromatic amino acid: tryptophan, tyrosine, or phenylalanine, and x can be any amino acid), and an acidic-Φ motif.

The Φ/Gly/Met-Φ/Gly/Met-x-P motif closely resembles the sequence of sorcin’s N-terminal peptide, which is located within a hydrophobic pocket formed by the D helix and the EF3 region. Trp105 and His108 within this pocket are likely critical for sorcin’s interaction with target proteins [7,9,12,13,14]. Sorcin interacts in a calcium-dependent manner with intrinsically disordered regions of target proteins, such as PPP1R3G, involved in glucose homeostasis [15].

Sorcin is a multifaceted player in cellular calcium homeostasis. Sorcin is a highly expressed calcium-binding protein and plays an important role in various processes, including cell division and survival.

Sorcin dynamically regulates calcium levels within the cell, particularly in the cytosol and in the endoplasmic reticulum (ER) and its associated vesicles [16].

## 3. Sorcin Regulates Several Physiological Processes

Sorcin acts in several ways, by interacting with and regulating several target proteins [17].

**Figure 2:**
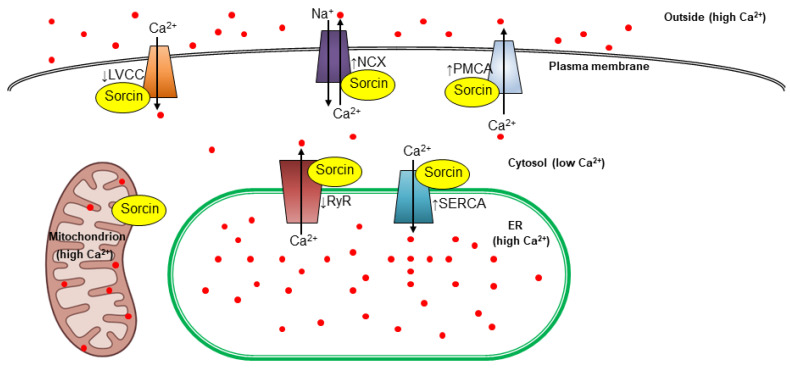
Sorcin regulates calcium homeostasis in the cells. Sorcin activates the activates the Na^+^-Ca^2+^ exchanger (NCX), the plasma membrane Ca^2+^ pump (PMCA) and modulates the L-type voltage-gated calcium channel (LVCC). In the endoplasmic reticulum (ER), sorcin inhibits ryanodine receptors (RyRs) and activates sarco/endoplasmic reticulum Ca^2+^−ATPase (SERCA), thereby increasing the Ca^2+^ load of the ER and decreasing ER stress (the arrows indicate Ca^2+^ flux). When sorcin expression is low, ER Ca^2+^ load (and also mitochondrial calcium load) is decreased, thereby increasing ER stress.

In particular, sorcin regulates calcium homeostasis by regulating channels, pumps, and exchangers (Figure 2). It inhibits calcium release by the ER, by binding to ryanodine receptors (RyRs), which are important channels that extrude calcium from the ER, acting as a brake on calcium release into the cell. In the heart, sorcin binds to cardiac RyRs with high affinity and completely inhibits channel activity. Sorcin significantly inhibits both the spontaneous activity of RyRs in quiescent cells and the inward Ca^2+^ current-triggered activity of RyRs that gives rise to Ca^2+^-induced Ca^2+^ release (CICR), i.e., the amplifying Ca^2+^ signaling mechanism that triggers cardiac contraction [18,19,20,21]. Sorcin also inhibits RyRs in endothelial and smooth muscle cells, effectively regulating Ca^2+^ signaling in vascular smooth muscle [22].

Furthermore, sorcin increases calcium uptake from the cytosol to the ER: sorcin increases the activity of the sarco/endoplasmic reticulum Ca^2+^ pump (SERCA), which actively transports calcium from the cytosol into the ER, further increasing ER calcium levels and reducing cytosolic ones [23].

Sorcin regulates other membrane calcium transporters: it activates the sarcolemmal Na^+^-Ca^2+^ exchanger (NCX), the plasma membrane Ca^2+^ pump (PMCA), and modulates the L-type voltage-gated calcium channel (LVCC) [14,24,25,26,27]. By regulating calcium transporters, sorcin plays a critical role in various cellular processes.

In heart muscle cells, sorcin helps regulate the contraction and relaxation cycle by controlling calcium fluxes [19,20]. Sorcin has a critical role in maintaining Ca^2+^ homeostasis, especially during the adrenergic response of the heart: in a sorcin knockout (KO) mouse model, sorcin deficiency can lead to ventricular arrhythmias and heart failure [28]. Sorcin KO hearts developed overexpression of LVCC and NCX, increasing spontaneous Ca^2+^ release events and leading to Ca^2+^ disturbances and arrhythmias. In turn, in vivo adenoviral transfer of sorcin reduces cardiac contractile abnormalities [29].

Additionally, sorcin also appears to modulate calcium handling of mitochondria: sorcin-overexpressing cardiac myocytes are more calcium-tolerant than wild-type cells and their mitochondria contain increased levels of calcium. Sorcin prevents cytochrome c release and caspase activation [30].

**Figure 3:**
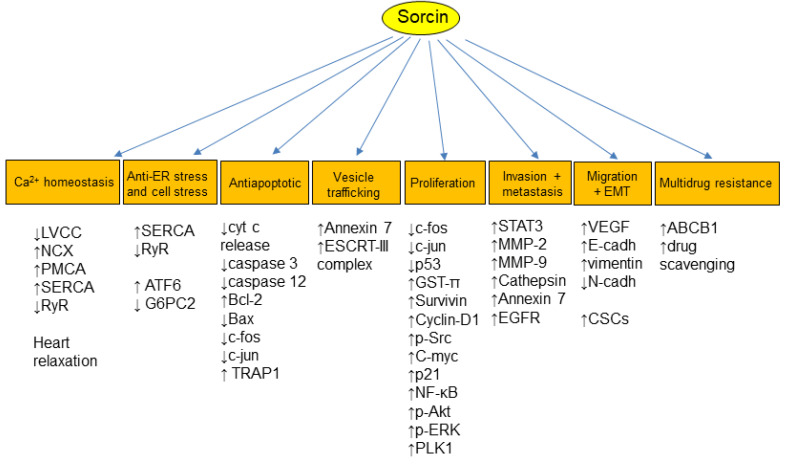
Sorcin regulates many cellular processes, by interacting with many proteins and/or regulating their activity or expression (see text). Sorcin participates in the regulation of calcium homeostasis, ER or cellular stress, apoptosis, vesicle trafficking, proliferation, invasion, metastasis, migration, epithelial-to-mesenchymal transition, and multidrug resistance. ↑: increased activity or expression; ↓: decreased activity or expression.

Sorcin calcium-dependently interacts with multiple proteins (Figure 3), including important kinases involved in the regulation of the cell cycle, such as Polo-like kinase 1 (PLK1), Aurora A, and Aurora B kinases. Sorcin physically interacts with PLK1, serving as a substrate for PLK1-mediated phosphorylation and stimulating PLK1 autophosphorylation, thereby regulating kinase activity. Sorcin is crucial for successful cell division, encompassing both mitosis and cytokinesis [16]. In fact, reducing sorcin levels leads to mitotic and cytokinetic abnormalities, an increase in abnormal, multinucleated cells, cell cycle arrest in the G2/M phase, and programmed cell death (apoptosis). Sorcin silencing triggers apoptosis by activating caspases 3 and 12 [16,30,31,32,33].

It is also worth mentioning that sorcin may help protect cells from ER stress, a condition caused by imbalanced calcium levels in the ER; sorcin overexpression can reduce apoptosis (programmed cell death) triggered by ER stress, by maintaining a high ER calcium concentration [16,30,32,33]. Sorcin overexpression activates ATF6, a key player in the unfolded protein response (UPR), which protects cells during ER stress, whereas sorcin KO reduces ATF6 transcriptional activity and increases ER stress. Notably, sorcin downregulation during lipotoxic stress may impair ATF6 activation and a normal UPR during the progression of obesity and insulin resistance [34].

Therefore, sorcin is emerging as a key player not only in UPR but also in cellular stress response and potentially in various pathologies. Sorcin interacts with carbohydrate-responsive element-binding protein (ChREBP), a key regulator of gene expression in pancreatic β cells and a significant contributor to glucose toxicity, sequestering it in the cytosol in a calcium-dependent fashion. Sorcin silencing inhibits ATP-induced increases in intracellular Ca^2+^ and glucose-stimulated insulin secretion. Sorcin may act as a Ca^2+^ sensor for glucose-induced nuclear translocation and the activation of ChREBP-dependent genes; a high carbohydrate diet enforces nuclear shuttling of hepatic NF-κB p65 and represses sorcin transcription, leading to de novo lipogenesis and intrahepatic lipid accumulation [35,36].

Sorcin overexpression in pancreatic beta cells increases ER Ca^2+^ levels and improves glucose tolerance and glucose-stimulated insulin secretion, highlighting its potential benefit in diabetes. Conversely, sorcin KO mice are glucose intolerant, with markedly impaired GSIS and increased expression of G6PC2, which contributes to lipotoxicity [33].

Another interesting aspect is the high sorcin expression in the brain, particularly in areas related to memory and emotion, which suggests its involvement in brain function [37]. Indeed, altered sorcin levels are observed in various neurodegenerative diseases, including Alzheimer’s disease (AD), Huntington’s disease, frontotemporal dementia, and Parkinson’s disease (PD). Interestingly, sorcin may offer protection against the rapid progression of these diseases. In neurons and microglia cells, sorcin colocalizes with ER proteins, such as RyRs, SERCA2, and the Sigma-1 receptor, and regulates ER calcium transients. Sorcin may represent both a novel early marker of neurodegenerative diseases and a response to cellular stress dependent on neurodegeneration [38,39,40,41,42,43,44,45,46,47,48].

Sorcin interacts with microtubule-associated tau, a protein that represents a pathological marker of AD. Binding to aberrant forms of tau impairs sorcin functions, such as calcium homeostasis and cellular resistance by ER stress, possibly resulting in a contribution to the progression of AD [49]. In addition, sorcin acts as an activator of PMCA, preventing the inhibitory effects of Aβ and tau on the pump; it also binds both Aβ and tau, counteracting their neurotoxicity [27].

Sorcin can interact with proteins like presenilin 2 (PS2) and alpha-synuclein (AS), implicated in AD and PD, respectively [50,51]. These interactions could influence both calcium signaling and disease development.

Furthermore, sorcin is involved in fertility. Sorcin is highly expressed in the endometrium, during the window of implantation, and decreased sorcin levels are observed in the endometrium of women with unexplained infertility. Sorcin is important in the regulation of Ca^2+^-mediated angiogenesis via the VEGF/PI3K/Akt pathway in endometrial cells and plays a crucial role in preparing the endometrium for implantation [52].

Sorcin is found in different types of vesicles within the cell, suggesting a potential role in cellular trafficking. High levels of sorcin have been identified in ER and in ER-derived vesicles positioned along microtubules. These vesicles are characterized by the presence of RyR, SERCA, calreticulin, and Rab10. Sorcin-containing vesicles are located at the mitotic spindle during mitosis, at the cleavage furrow during telophase, and finally at the midbody. Sorcin regulates the size and calcium content of ER vesicles by decreasing RYR activity and stimulating SERCA function [16]. Sorcin is found at high levels in lipid rafts and in exosomes [53,54,55,56,57,58,59,60,61,62,63,64]. Further, endosomal recruitment of sorcin was identified as an essential step in apoptotic exosome formation since it participates with annexins A2 and A7 and the HD-PTP protein in the assembly of the ESCRT-III complex [63].

## 4. Sorcin and Cancer

Sorcin is often overexpressed in cancer cells, in many types of tumors, such as neuroblastoma, adenocarcinoma, glioma, ovarian cancer, gastric cancer, lung cancer, oral cancer, prostate cancer, colorectal cancer, central nervous system cancer, gallbladder cancer, hepatocellular carcinoma, and leukemias [32,65,66,67,68,69,70,71,72,73,74,75,76,77,78,79,80,81,82,83,84,85,86,87,88,89,90].

A recent study investigated the expression of sorcin in normal tissues vs. cancer tissues using multiple databases, i.e., Genotype-Tissue Expression (GTEX), The Cancer Genome Atlas (TCGA), and Oncomine [91]. The analysis showed that sorcin is significantly overexpressed in 25 types of cancer (it is also downregulated in 3 cancers; no significant difference is reported for 5 cancers), and that sorcin expression dysregulation could predict worse prognosis in the patients with many types of cancer. The same study reports a correlation of sorcin expression with patient survival, clinical features, cancer stem cell properties, tumor mutation burden, microsatellite instability, and immune cell infiltration; aberrant sorcin expression and/or gene amplification was also found related to epithelial-mesenchymal-transition (EMT), tumor immune microenvironment, and drug resistance (see below) [91].

Sorcin overexpression seems to contribute to cancer cell survival by means of different features. Ca^2+^ signaling plays a crucial role in regulating many cancer hallmarks, such as angiogenesis, migration, and invasion; abnormal calcium fluxes due to altered channel/pump expression or activation participate in carcinogenesis and promote cancer development and resistance to chemotherapeutic drugs (for a review, see reference [92]). Sorcin contributes to the regulation of LVCC, NCX1, PMCA, SERCA, and RyRs (Figure 2), and its overexpression leads to calcium dyshomeostasis. As already mentioned, sorcin can help cancer cells evade apoptosis by modulating calcium homeostasis and, in particular, increasing calcium levels in the ER and mitochondria, thereby reducing ER stress and cellular stress [16,30,32,33,93].

Sorcin modulates cell cycle and apoptosis, also by regulating important regulation pathways (Figure 3). Leukemia and breast carcinoma cells overexpressing sorcin also showed high expression of Bcl-2, along with decreased level of Bax [75,94]; upon sorcin silencing, cells show increased Bax, c-fos and c-jun expression, while the level of Bcl-2 is decreased [94]. In nasopharyngeal carcinoma, sorcin silencing results in the expression of the multidrug-resistance genes MDR1 (ABCB1), MRP1, and ERCC1, the detoxification enzyme GST-π, the Rho GTPase RhoE, and the anti-apoptotic proteins Bcl-2 and Survivin. Conversely, PTEN expression increased, while the levels of phosphorylated Akt (p-Akt) and NF-κB decreased, leading to reduced apoptosis [95]. Similarly, in myeloma cells, sorcin silencing results in reduced cell proliferation, cell cycle arrest, and cell apoptosis, with reduced expression levels of ABCB1, MRP1, GST-π, Survivin, Livin, Bcl-2, Cyclin-D1, phospho-Src, C-myc, p21, NF-κB and phospho-AKT, while p53 expression and caspase-3 and caspase-8 activity significantly increased [96]. Sorcin silencing also activates GRP78/BiP and caspases 3 and 12, triggering apoptosis via the mitochondrial pathway [32].

Sorcin interacts with various other proteins involved in cell cycle regulation and signaling pathways, such as Polo-like kinase 1 (PLK1), Aurora A and Aurora B kinases. Sorcin silencing results in defects in mitosis and cytokinesis and in increased apoptosis [16]. Sorcin interacts with the calcium/calmodulin-dependent protein kinase II (CaMKIIdeltaC), inhibiting it in a calcium- and concentration-dependent manner [97].

TRAP1, a mitochondrial anti-apoptotic heat shock protein, involved in protection from oxidative stress and apoptosis, specifically interacts with sorcin in a Ca^2+^-dependent fashion; this interaction is required for the anti-apoptotic function of TRAP1 [98].

Sorcin is an important player in angiogenesis, proliferation, EMT and tumor metastasis (Figure 3). Sorcin silencing decreases the expression of the angiogenic factor VEGF and of its downstream effector molecules, i.e., PI3K, Akt, and NOS. Sorcin regulates Ca^2+^-mediated angiogenesis via the VEGF/PI3K/Akt pathway [52].

Sorcin overexpression increases EMT, migration, and invasion in cancer cells, while sorcin silencing reduces the pool of cancer stem cells (CSCs), decreasing the EMT transition and suppressing metastases via the reduced expression of E-cadherin and VEGF and by the PI3K/Akt axis [81,99]. Sorcin knockdown decreases the expression of matrix metalloproteinases (MMP2 and MMP9), cathepsin Z, and STAT3, leading to suppressed tumor growth and metastasis [100]. In ovarian cancer, sorcin was found to drive malignant progression via the Smad4/ZEB1/miR-142-5p axis [86]. The interaction between sorcin and Annexin 7, both overexpressed in hepatocellular carcinoma, is able to promote EMT, migration, invasion, and proliferation, contributing to tumor aggressiveness [101].

Sorcin and EGFR expression levels are significantly correlated and linked to decreased overall survival in cancer patients. Sorcin directly interacts with the EGFR protein in a calcium-dependent manner, regulating calcium balance associated with EGFR signaling induced by EGF. Sorcin influences EGFR protein stability and signaling, enhancing its phosphorylation, and subsequently promoting EGF-driven cell migration and invasion. Notably, suppressing sorcin expression enhances the effectiveness of EGFR inhibitors in controlling cell migration [102].

In ovarian cancer, sorcin overexpression is associated with an impaired TGF-β signaling pathway. TGF-β1 administration, in fact, inhibits sorcin expression [103].

Sorcin not only regulates apoptosis but also pyroptosis. High sorcin expression in hepatocarcinoma cells negatively regulates pyroptosis by interacting with the NLRP3 inflammasome to promote tumor proliferation, migration, and invasion [88].

## 5. Multidrug Resistance

Multidrug resistance (MDR) is the main reason why many cancers become resistant to chemotherapy drugs. This significantly reduces the effectiveness of many chemotherapy treatments. It affects patients with a wide range of blood cancers and solid tumors. Tumors are often composed of a mix of malignant cells, with some being sensitive to drugs while others are resistant. Chemotherapy eliminates the drug-sensitive cells, but this leaves behind a larger proportion of resistant cells. When the tumor starts to regrow, chemotherapy may fail because the remaining cells are now drug-resistant. Over 90% of metastatic cancers exhibit MDR, leading to treatment failure [104].

**Figure 4:**
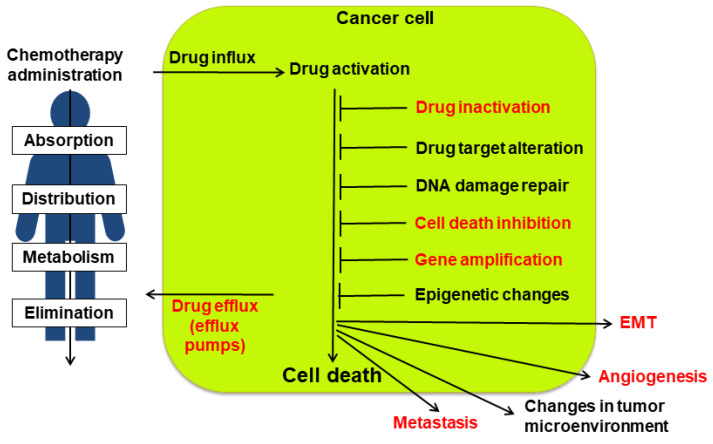
Upon administration of chemotherapeutic drugs, many factors determine multidrug resistance (MDR). These factors include absorption, distribution, metabolism, and elimination (ADME), drug influx, drug efflux by efflux pumps, drug activation and inactivation, drug target alteration, DNA damage repair, the inhibition of cell death, and in particular of apoptosis, gene amplification, epigenetic effects, epithelial-to-mesenchymal transition (EMT), angiogenesis, changes in tumor environment, and metastasis. Sorcin participates in several mechanisms of MDR onset (indicated in red, see text).

A multitude of both intrinsic and acquired mechanisms contribute to drug resistance in cancer (Figure 4) (for a review, see [104,105,106]). The efficacy of chemotherapy is hindered by factors such as poor drug solubility, toxicity to healthy tissues, and pharmacokinetic limitations on drug delivery to the tumor.

At the tumor level, diverse resistance mechanisms emerge, including impaired drug uptake, enhanced drug efflux, altered cellular composition, inhibited cell death, increased DNA repair, cell cycle abnormalities, drug sequestration, accelerated drug metabolism, drug target modifications, and epithelial-mesenchymal transition (EMT). Some of these mechanisms confer resistance to specific drugs, while others contribute to MDR, rendering tumors refractory to multiple treatments and significantly reducing cure rates.

Further, tumor heterogeneity, driven by genetic and non-genetic factors, fuels the development of drug resistance. Clonal selection during therapy favors the survival and expansion of resistant cancer cell subpopulations. Notably, cancer stem cells (CSCs) play a pivotal role in chemoresistance due to their intrinsic resistance, mediated by epigenetic factors, increased expression of anti-apoptotic proteins and drug efflux pumps, and their dormant state. Consequently, chemotherapy primarily eliminates sensitive cancer cells, allowing resistant subpopulations, including CSCs, to proliferate and cause treatment failure.

## 6. Sorcin in Multidrug Resistance

Sorcin was initially discovered as a marker of MDR, although it is increasingly considered a cause of MDR. Sorcin was initially discovered in vincristine-resistant hamster lung cancer cells, and it is often overexpressed in cancers exhibiting an MDR phenotype [31,32,65,66,73,75,78,80,82,83,84,86,91,95,96,103,107,108,109,110,111].

Sorcin transfection in several tumor cell lines (leukemia, lung, gastric, ovarian, and breast cancers) increases resistance to common chemotherapy drugs, e.g., paclitaxel, doxorubicin, vincristine, etoposide, 5-fluorouracil and homoharringtonine [73,75,80,84,86,94,109,110,111].

Conversely, silencing sorcin increases chemotherapeutic drug toxicity and reverses MDR in various cancers, including leukemia, cervical cancer, breast cancer, colorectal cancer, and nasopharyngeal carcinoma [31,32,73,78,82,83,84,86,94,95,103,107,108,109,112,113].

Sorcin’s influence on ABCB1 (MDR1 or P-glycoprotein) is perhaps the most well-studied and significant mechanism of MDR it contributes to. ABCB1, an ATP-dependent efflux pump, is a critical factor in resistance to a broad spectrum of chemotherapy drugs, including anthracyclines, taxanes, and Vinca alkaloids, in various cancers (solid and blood-based tumors) [114,115]. Its overexpression or amplification significantly reduces the effectiveness of these drugs, increasing their efflux from cancer cells.

Interestingly, the genes for sorcin and ABCB1 (the most significant ATP-dependent drug efflux pump) are located near each other on chromosome 7 and can be co-amplified, leading to their simultaneous overexpression in MDR cancer cells [3,65].

For a long time, sorcin overexpression was thought to be only an accidental consequence of this co-amplification. However, in the last two decades, many studies showed that sorcin is not just a marker but also a cause of MDR, since it contributes to many processes leading to resistance (Figure 4).

Sorcin plays a key role in regulating ABCB1 expression. Sorcin overexpression leads to increased ABCB1 levels, ultimately enhancing drug resistance in various cancer cell lines, including those from gastric cancer, lung tumors, nasopharyngeal carcinoma, cervical carcinoma, and leukemias, while sorcin silencing reduces ABCB1 expression [31,80,83,95,96,109,110,111,116].

Mechanistically, sorcin stimulates the phosphorylation of CREB1 by PKA (protein kinase A). This activated CREB1 then binds to the cAMP response element (CRE) within the ABCB1 gene promoter, located at −716 to −709 bp, leading to increased ABCB1 expression [80]. Conversely, sorcin silencing can suppress ABCB1 expression by inhibiting ERK and Akt signaling pathways [111].

Interestingly, the genes for sorcin (SRI) and ABCB1 reside within the same chromosomal region (7q21.12) in both humans and mice. Chromosomal amplification events at the 7q21 locus lead to marked ABCB1 overexpression, which in turn causes MDR (for a review, [115]). This shared location suggests a potential link between their co-amplification, where both genes are duplicated together. Amplification of this region is observed in many multidrug-resistant cancers, leading to the overexpression of both sorcin and ABCB1, further contributing to MDR [3,77,117,118,119,120,121,122,123,124,125,126,127,128,129,130,131].

The amplicon at the 7q21.12 region encompasses several other genes beyond sorcin and ABCB1. Many of these genes, including ABCB4 (MDR3), ADAM22, and DBF4, have been implicated in cancer development and MDR, and are considered possible therapeutic targets [132,133,134,135,136,137,138,139,140,141,142,143,144,145,146].

Sorcin’s contribution to a multidrug-resistant phenotype may also involve its ability to directly bind chemotherapeutic drugs (Figure 4). Studies using techniques like surface plasmon resonance and X-ray diffraction demonstrate sorcin’s high-affinity binding to doxorubicin, paclitaxel, vincristine, and cisplatin in vitro [110]. The crystal structure of the sorcin-doxorubicin complex revealed one of the binding sites—near the interface of two sorcin monomers. This binding pocket involves interactions between specific sorcin residues and the doxorubicin molecule [108]. Additionally, sorcin’s cellular localization changes upon doxorubicin treatment, suggesting potential drug interactions within the cell. These findings suggest sorcin could act as a “drug scavenger,” limiting the toxic effects of doxorubicin and potentially other drugs within the cell [110].

## 7. Targeting Sorcin: Promising Strategies for Overcoming Cancer and MDR

Sorcin’s involvement in tumorigenesis and MDR across various cancer cell lines suggests that lowering its expression could be a viable strategy to reverse both cancer onset and resistance to chemotherapeutic targets. This has led to the exploration of several promising approaches targeting sorcin (Table 1).

MicroRNAs (miRNAs) act as natural gene regulators. miR-1, for example, targets the 3′ untranslated region (3′-UTR) of the sorcin gene (*SRI*) and modulates calcium transients in heart muscle cells. Interestingly, miR-1 expression is downregulated in human heart failure and corresponding mouse models [147]. In MDR gastric cancer cells, miR-1 levels are significantly low with respect to non-tumor tissues. Reintroducing miR-1 promotes apoptosis (programmed cell death), inhibits migration, and increases the accumulation of chemotherapeutic drugs like doxorubicin and vincristine by acting on sorcin expression [112,148]. While sorcin overexpression can partially counteract miR-1’s effect, this finding supports the potential of miR-1 as a therapeutic strategy against sorcin-dependent MDR. However, off-target effects are a concern, as miR-1 regulates other genes like HSP60 (involved in diabetic heart damage) and the ets1 proto-oncogene (important for extracellular matrix breakdown) [149,150]. In ovarian cancer, miR-142-5p suppresses sorcin expression by binding to the 3′-UTR of the *SRI* gene; interestingly, this is part of a complex homeostatic loop, since the transcription factor ZEB1 inhibits the transcription of miR-142-5p by directly binding to the E-box fragment in the miR-142 promoter region, while furthermore, ZEB1 is itself negatively regulated by sorcin [86].

Many molecules have effects on sorcin levels and may represent options to control tumorigenesis, MDR, and other pathological conditions (Table 1).

Since sorcin overexpression in ovarian cancer is associated with an impaired TGF-β signaling pathway, this pathway may represent a target to regulate sorcin expression. In fact, the administration of TGF-β1 inhibits sorcin expression in ovarian cancer and non-small cell lung cancer [103].

Tetrandrine, a bis-benzylisoquinoline alkaloid, is a well-known calcium channel blocker, which inhibits voltage-gated Ca^2+^ current (LVCC) and Ca^2+^-activated K^+^ current. Tetrandrine decreases sorcin expression and reverses MDR in leukemia cell lines resistant to daunorubicin, in a concentration-dependent fashion [151].

The oxindole derivative PH II-7 is a powerful cytotoxic agent, able to increase intracellular drug concentration in multidrug-resistant cells by inhibiting the expressions of both ABCB1 and sorcin, and to induce the apoptosis of multidrug-resistant tumor cells [152].

Dihydromyricetin (DMY), a natural compound with anti-oxidant, anti-inflammatory, and anti-tumor properties, has shown promise in reversing MDR in leukemia and breast cancer cells resistant to adriamycin, as well as in animal models. It increases adriamycin cytotoxicity by decreasing sorcin (both mRNA and protein) and, consequently, ABCB1 levels, likely via the ERK/Akt signaling pathway [84,111]. Additionally, DMY increases intracellular-free calcium levels, reactive oxygen species (ROS), and caspase 12 expression (markers of ER stress-induced apoptosis). It also regulates markers of mitochondrial apoptosis, such as caspases 3 and 9, Bcl-2, Bax, and PARP [84].

Ondansetron (OND), an anti-emetic drug used during chemotherapy, has also been shown to reverse MDR associated with sorcin expression, particularly when combined with DMY. This combination increases cell cycle arrest and apoptosis, possibly by decreasing MDM2/MDMX, and consequently increasing p53 function. Both DMY and OND may act by binding sorcin with high affinity, although DMY’s effect seems specific and dependent on sorcin binding [111].

Haishengsu (HSS) is a protein extract, produced by *Tegillarca granosa* seashells. HSS administration reduces sorcin, BCR/ABL, and ABCB1 expression in mouse models and results in increased apoptosis in MDR leukemia cells. While its effects on other targets remain unclear, HSS (added to conventional chemotherapy) has shown promising results in clinical trials for acute leukemia patients, increasing treatment efficacy and improving quality of life [153,154].

Calcitriol (vitamin D) binds to the vitamin D receptor, which acts as a transcription factor regulating various cellular processes, including sorcin expression. Calcitriol treatment leads to differentiation and anti-proliferative effects and appears to increase the 19 kDa form of sorcin, suggesting potential for further investigation [155].

Palmitate, a free fatty acid, downregulates sorcin in pancreatic beta cells, potentially contributing to lipotoxicity and ER stress. In addition, palmitate treatment reduces phosphorylation of AMPK and increases the levels of markers of the proapoptotic arm of ER stress response (pEIF2α and CHOP) and apoptosis (cleaved caspase 3). Interestingly, metformin treatment normalizes the levels of pAMPK, pEIF2α, CHOP, and cleaved caspase 3 and increases the levels of sorcin; palmitate controlling may thus represent a treatment strategy aiming at decreasing insulin levels in obese children [156,157].

TNFα decreases sorcin expression in pancreatic cells, possibly by increasing phosphorylation of key proteins of insulin secretion pathway such as mTOR and NFκB; fractalkine (CX3CL1) restores sorcin levels by reducing mTOR expression and decreasing phosphorylation of mTOR and NFκB [158].

Triptolide, a diterpenoid epoxide from the thunder god vine, *Tripterygium wilfordii*, has proapoptotic activity and anti-tumor effects in ovarian cancer cells, reducing the expression of sorcin, MMP-2, and VEGF, which are usually highly expressed in ovarian cancer cells [83]. Since triptolide is rather toxic, strategies aimed at specifically delivering it to cancer cells have been developed. The administration of triptolide-loaded exosomes (which have high drug encapsulation efficiency and a rather specific ability to deliver their payload to cancer cells) had a high capacity of inhibiting ovarian tumor cell proliferation and tumor growth, although they had toxic effects on liver and spleen [159].

A more direct way to target sorcin is the use of CRISPR or siRNA vs. sorcin to restore calcium homeostasis, limit tumorigenesis, and overcome chemoresistance. siRNAs inhibit target gene expression by binding to specific mRNA transcripts and proved to be effective in cancer cell cultures (see Section 4 and Section 5); however, siRNA is limited in clinical application due to its nature, such as low internalization in tumor cells, degradation by enzymes in the bloodstream, and off-targeting features. Loading siRNAs into nanocarriers protects them from nuclease degradation, increases their delivery to tumors by the enhanced permeability and retention (EPR) effect, and decreases siRNA-dependent immune stimulation. Lipid-coated albumin-paclitaxel nanoparticles loaded with sorcin-siRNA (LANP-PTX-siSRI) have been recently developed for PTX and siSRI co-delivery, aiming at sorcin targeting and PTX delivery into multidrug-resistant cells. The LANP-PTX-siSRI decreased sorcin expression and increased intracellular calcium, leading to the inhibition of PTX-resistant ovarian cancer cell growth [103].

## 8. Conclusions

Sorcin’s co-amplification with drug efflux pumps and its role in calcium signaling make it a fascinating target for cancer therapy. Extensive research has been dedicated to unraveling sorcin’s mechanisms of action in various disease contexts. This knowledge paves the way for the development of novel and effective therapeutic strategies.

Developing effective sorcin-targeting therapies is complex due to sorcin’s widespread expression in healthy tissues. Off-target effects and potential for resistance development are major concerns, similar to many chemotherapeutic drugs. Recent advancements in targeted therapies, such as antibody-drug conjugates and gene editing techniques, offer promising avenues for overcoming these challenges.

A key future direction lies in acquiring a deeper understanding of sorcin’s protein interactions (interactome). This knowledge could help identify specific interaction sites for targeted modulation, minimizing off-target effects. Additionally, structural studies suggest that calcium binding exposes specific hydrophobic regions on the sorcin molecule. These regions could potentially serve as targets for interaction with other proteins. By targeting these specific sites, researchers may achieve focused modulation of sorcin’s activity, leading to more effective and specific therapeutic strategies.

## 9. Perspectives

Sorcin is an important protein, involved in many cellular processes.

It is essential for ER calcium signaling, controlling ER size, calcium content, and vesicle dynamics by inhibiting RyR and activating SERCA. Upon calcium binding, sorcin undergoes conformational changes exposing hydrophobic residues, enabling interactions with (and regulation of) calcium channels and exchangers like RyR and SERCA. This modulation increases ER calcium accumulation, mitigating ER stress and the UPR. Possibly because of this action on ER stress, sorcin is also an early marker of neurodegeneration.

In particular, sorcin influences various oncogenic proteins, including NF-κB, STAT3, AKT, ERK1/2, IP3R, VEGF, MMPs, and caspases, and drug efflux pumps such as ABCB1. Its suppression induces apoptosis, mitotic and cytokinetic defects, cell cycle arrest, and the formation of abnormal cells.

Overexpressed in multiple cancers, especially chemoresistant ones, sorcin promotes proliferation, migration, invasion, EMT, and drug resistance. Conversely, its inhibition sensitizes cells to cisplatin and adriamycin, reducing proliferation and inducing apoptosis.

Given its oncogenic properties and association with MDR, sorcin is considered an oncogene and an MDR marker.

Further, it is increasingly considered a potential therapeutic target since its regulation, by means of several categories of molecules, can affect cancer and/or resistance to chemotherapeutic drugs.

## Figures and Tables

**Table 1:** Experimental approaches targeting sorcin expression. Sorcin expression was shown to be selectively decreased by using microRNAs (miR-1, miR-142-5p), siRNAs (in nanocarriers), growth factors (TGF-β1, TNFα), small molecules (Tetrandrine, PH II-7, Dihydromyricetin, Ondansetron, Calcitriol, Palmitate, Triptolide) and protein extracts (Haishengsu). The chemokine fractalkine restores sorcin expression decreased by TNFα.

Category of Molecules	Molecule	Mechanism of Regulation	Effect
MicroRNAs	miR-1	Binding to 3′-UTR of *SRI* gene	↓ sorcin
	miR-142-5p	Binding to 3′-UTR of *SRI* gene	↓ sorcin
siRNAs	siRNA vs. sorcinin nanocarriers	Binding to sorcin mRNA	↓ sorcin
Growth factors	TGF-β1	↑ SMAD2 phosphorylation	↓ sorcin
	TNFα	↑ mTOR, NFκB phosphoryl.	↓ sorcin
Chemokine	Fractalkine	↓ mTOR, NFκB phosphoryl.	↑ sorcin
Small molecules	Tetrandrine	Calcium channel blocking	↓ sorcin
	PH II-7	?	↓ sorcin
	Dihydromyricetin	↓ ERK-Akt phosphorylation	↓ sorcin
	Ondansetron	Sorcin binding	↓ sorcin
	Calcitriol	?	↓ sorcin
	Palmitate	↓ AMPK- pEIF2α phosphoryl.	↓ sorcin
	Triptolide	?	↓ sorcin
Protein extracts	Haishengsu	?	↓ sorcin

↑: increased activity or expression; ↓: decreased activity or expression; ?: unknown.
